# Perspectives on weight gain and lifestyle practices during pregnancy among women with a history of macrosomia: a qualitative study in the Republic of Ireland

**DOI:** 10.1186/1471-2393-13-202

**Published:** 2013-11-06

**Authors:** Emily Heery, Áine McConnon, Cecily C Kelleher, Patrick G Wall, Fionnuala M McAuliffe

**Affiliations:** 1 School of Public Health, Physiotherapy and Population Science, University College Dublin, Dublin, Ireland; 2 UCD Obstetrics and Gynaecology, School of Medicine and Medical Science, University College Dublin, National Maternity Hospital, Dublin, Ireland

**Keywords:** Weight gain during pregnancy, Macrosomia, Qualitative research, Lifestyle practices

## Abstract

**Background:**

Excessive weight gain during pregnancy is a major risk factor for macrosomia (high birth weight delivery). This study aimed to explore views about weight gain and lifestyle practices during pregnancy among women with a history of macrosomia.

**Methods:**

A qualitative descriptive study was conducted. Twenty-one second-time mothers whose first infant was macrosomic (>4 kg) were recruited from a randomised trial in a large maternity hospital in the Republic of Ireland. Semi-structured interviews were conducted with participants at both 6 and 12 months after their second pregnancy. Inductive thematic analysis was used to identify distinct themes.

**Results:**

The mothers believed in following their prenatal food cravings to meet their baby’s needs, but this led some to eat excessively. Many of the women cut back heavily on physical activity during pregnancy due to perceived risks to the baby. Physical conditions and discomforts during pregnancy often limited maternal control over weight and lifestyle practices. The women were not particularly concerned about weight gain during pregnancy and most did not favour the notion of introducing weight gain guidelines into routine antenatal care. Common differences perceived by the women between their first and second pregnancy included: increased concern about weight gain in their second pregnancy due to prior difficulties with postpartum weight loss and increased time demands in their second pregnancy impeded healthy lifestyle practices. Most women did not alter their perspectives on weight gain and lifestyle practices in their second pregnancy in response to having a macrosomic infant in their first pregnancy.

**Conclusions:**

This analysis exposed numerous barriers to healthy pregnancy weight gain. The findings suggest that women may need to be advised to follow their prenatal food cravings in moderation. Pregnant women with children already may benefit from education on time-efficient methods of integrating healthy eating practices and physical activity into their lifestyles. Women with a history of macrosomia may need information about the importance of avoiding high weight gain in subsequent pregnancies.

## Background

High proportions of pregnant women are gaining weight above the Institute of Medicine’s recommended ranges [1]. Indeed, the prevalence of excessive gestational weight gain ranged between 49 and 64% in five recent studies conducted in industrialised countries [2-6]. There is consistent evidence that regardless of pre-pregnancy BMI, excessive prenatal weight gain confers a greater risk for macrosomia (high birth weight), caesarean delivery and postpartum weight retention [7-9]. The negative outcomes of excessive prenatal weight gain highlight the need for weight management interventions to promote healthy body weight changes during the childbearing years. An in-depth understanding of women’s perceptions about weight management during pregnancy is needed to inform the design of such interventions.

Women’s perspectives on weight gain during pregnancy have been explored in a wide range of subpopulations, including low-income, overweight and ethnic minority groups, but not among women with a history of macrosomia [10-13]. High infant birth weight has been associated with an increase in adverse maternal and child outcomes, including delivery complications and childhood obesity [14-16]. Historically, obstetric concerns about heavy babies were primarily focused on diabetic pregnancies [17]. However, in the last 3 decades, the overall proportion of women delivering macrosomic infants has increased in many countries [18-22]. Investigators have shown that newborn macrosomia recurs in a second pregnancy in about one-third of cases and that gestational weight gain influences this risk [23,24]. Understanding perspectives on prenatal weight gain among women with a history of macrosomia may enable health professionals to assist women in achieving or maintaining a healthy weight during the childbearing years and may also inform future strategies to prevent macrosomia. Consequently, the present study used semi-structured interviews to explore the views of second-time mothers whose first infant was macrosomic about weight gain and lifestyle practices during pregnancy.

## Methods

This is a qualitative study with institutional ethical approval and written informed consent. Ethical approval was obtained from the Ethics Committee of the National Maternity Hospital, Dublin. The present analysis explored perspectives on weight gain and lifestyle practices during pregnancy among second-time mothers with a history of macrosomia. This research was guided by an interpretive and descriptive approach that enables researchers to examine phenomena in depth from the standpoints of the individuals involved [25]. In-depth, semi-structured interviews were used to elicit a detailed understanding of women’s views and experiences in a flexible and responsive manner [26].

The interview sample was recruited from the ROLO study, a randomised trial of a low glycaemic index diet in pregnancy to prevent macrosomia in women with a history of macrosomic delivery [27]. Second-time mothers whose first infant was macrosomic (>4 kg) were recruited from the control group of the ROLO study. The women included in the qualitative study upon entry to antenatal care in their second pregnancy were aged 18 years or above, had a singleton pregnancy and did not have a history of diabetes or other medical disorder. Furthermore, purposive sampling was utilised to recruit mothers who varied in terms of BMI measured in the first trimester of their second pregnancy. Recruitment was based on BMI measured in the first trimester rather than pre-pregnancy BMI, as pre-pregnancy measured weight was unavailable and self-reported pre-pregnancy weight tends to be underestimated, particularly among heavier individuals [28,29]. Furthermore, weight gain in the first trimester is known to be small [1].

The first author approached eligible women at their 6-month postpartum research appointment for the ROLO study at the National Maternity Hospital, Dublin, about taking part in the qualitative study. Each woman was given an information sheet which detailed what participation involves, her rights as a participant and what will happen with the data collected. Recruitment was completed when no new relevant information was acquired from the interviews which indicated that data saturation was reached.

As per the control arm of the ROLO study, the recruited women received routine antenatal care. As is standard practice at the National Maternity Hospital, this did not involve any formal advice about body weight, gestational weight gain or diet [27].

An experienced female researcher (first author) conducted in-depth interviews with participants on two occasions: at 6 and 12 months after their second pregnancy. The interview guides covered three main areas: 1) lifestyle practices (e.g., diet, physical activity and infant feeding); 2) weight management (e.g., advice received and body image) and 3) well-being (e.g., stress and emotional and physical well-being). The first interview explored women’s perspectives on weight and lifestyle practices before having children, during their first and second pregnancy and in the 6-month period since having their second child. The interview guide for the first interview is included as Additional file 1. The second interview explored women’s views about weight and lifestyle practices in the year following their first and second pregnancy. Although the second interview focused on the postpartum period, both interviews provided data for the present analysis as the women often referred to their prenatal experiences when answering questions about the postpartum period.

A short questionnaire was given to each of the participants at the end of the first interview to collect demographic information. Following each interview, field-notes describing the interview setting, the body language of the participants and any other initial impressions were written up in a research diary; this information was collected to provide a richer understanding of the interview situation and thus aid data analysis [30,31].

Each interview was recorded on a digital recorder and then saved onto a password protected PC. The interview was then deleted from the recording device. The interviews were transcribed verbatim by an external company, experienced in transcription of qualitative interviews. Following transcription, the interview transcripts were checked against the recordings for accuracy and then anonymised.

Inductive Thematic Analysis was used to analyse the transcripts and accompanying field notes [32]. Basic codes were applied across the entire dataset for all patterns of meaning relevant to the research question. A mind map [33] was then developed by organising the codes into a hierarchal system of themes and subthemes. The development of the thematic map and the interpretation of findings were discussed and refined during meetings of the research team. A qualitative data analysis program (NVIVO 9) was used to aid the storage and arrangement of the themes and subthemes.

To enhance the trustworthiness of the research, initial impressions from the first interview were discussed with the interview participants at the end of the second interview as a form of member check [34,35]. Additionally, the first author had regular meetings with a colleague who was independent of the research team to validate the coding process [34,35]. During the initial coding phase, a sample of interviews was coded independently by both researchers. Any discrepancies between the two sets of coding were discussed until a consensus was reached.

## Results

Twenty-one women participated in this study between November 2009 and December 2010. Twenty-one women participated in an interview at 6 months postpartum and 18 of these participated in a second interview at 12 months postpartum. All but one of the interviews was conducted in the women’s own homes. The interviews at 6 months postpartum lasted between 30 and 80 minutes. The interviews at 12 months postpartum lasted between 25 and 70 minutes.

The women ranged in age from 23 to 41 with a mean age of 32 years. All of the women were Caucasian and four of the women were born outside of Ireland (one was German, one was Ukrainian and two were English). The mean BMI of the sample at their 'booking’ visit in their second pregnancy was 26.3, which is overweight. Nine of the mothers were normal weight, eight were overweight and four were obese. Most were in paid employment (19 women) and approximately two-fifths (eight women) had graduate degrees. We compared the interview sample with the ROLO study sample and found that it was representative of the larger study across a range of demographic characteristics including age, educational attainment and smoking status.

Four distinct themes were identified from the thematic analysis which explored women’s perspectives on weight gain and lifestyle practices during pregnancy:

1) An exceptional time for lifestyle practices

2) Managing the home and work environment

3) Bodily changes

4) Weight consciousness

The participants’ names and any other identifying information were changed to protect the participants’ anonymity.

### An exceptional time for lifestyle practices

The first theme consists of two subthemes: 1) following bodily calls and 2) the risks or rewards of physical activity in pregnancy. The mothers viewed pregnancy as an exceptional time for lifestyle practices, when the baby’s needs have to take prominence over their own needs.

#### *Following bodily calls*

The women generally described an increased awareness of healthy eating habits during pregnancy and recalled making an effort to eat more healthy foods, except if they perceived their diet to be very healthy already. All of the women believed in following their bodily 'calls’ or cravings during pregnancy, as this was seen as the best way of meeting their baby’s nutritional needs. Most of the women were also aware that you are not supposed to follow the traditional adage of 'eating for two’ during pregnancy, but in spite of this knowledge, some recalled using pregnancy as an 'excuse to indulge’ , especially in their first pregnancy. This often involved eating large amounts of treats, such as chocolate and biscuits, which they would only have consumed in small amounts before pregnancy. Some described their eating during their pregnancies as uncontrollable and excessive, as they ate everything they wanted, or craved:

I didn’t particularly watch what I ate during my [second] pregnancy, I kind of just ate what I wanted, when I wanted, you know, and I suppose naively you feel it’s a chance to or a time to indulge yourself, rightly or wrongly. (Cliona)

I think like the first time around I was like, 'well I can’t help it, you know, I’m pregnant, this is what my body wants’. (Silvia)

Over-indulgence during pregnancy often led to feelings of guilt, as suggested by descriptions of foods eaten as 'bad’ or 'bold’. This guilt appeared to arise because the women wanted to fulfil all of their food cravings or wants and thus meet their baby’s needs, but this contradicted their knowledge about the importance of healthy eating and not 'eating for two’ during pregnancy:

I did try. I was eating healthy [in the second pregnancy], but then eating bold stuff as well. (Lisa)

I kind of think it is nearly an excuse in a way - 'you can eat whatever you want really’. Well you can’t, but you do - I did. Lots of people don’t. I used to when I was pregnant with Liza [first child], I used to buy chocolate all the time – it was an excuse to gorge on chocolate and ice-cream and all those things. (Clare)

Pre-pregnancy lifestyle habits were often perceived to influence food cravings during pregnancy. A number of women perceived that, as they had to give up a lot of things during pregnancy, such as smoking and drinking, they compensated with food. In particular, smoking cessation was perceived to lead to increased cravings for 'junk’ foods:

So, I gave up smoking when I was pregnant with her [first child] and maybe that was why I didn’t eat as much chocolate before…I just craved chocolate more I guess. (Clare)

Some participants also perceived that women who restricted their eating before pregnancy often ate excessive amounts during pregnancy:

But like I said a lot of people who would maybe not eat enough when they’re not pregnant go all hell for leather when they are. (Susan)

In contrast, some of mothers perceived that they did not 'eat for two’ during their pregnancies, but instead followed their wants and cravings in moderation. Many of these mothers emphasised the importance of listening to your body and continuity in healthy eating, before and throughout pregnancy:

You see, some women, they, they are worried about the baby; the baby has to grow…so some people try to eat more…which I think is not necessary. You eat just as you ate before, just a little bit more - some extras that you want. (Caroline)

Many of the mothers who described following their bodily calls in moderation mentioned eating treat foods as well as healthy foods during pregnancy, but this was not a source of guilt for the mothers and was instead depicted as a normal, balanced diet. In the following excerpt, a participant described balancing healthy foods with treat foods in both of her pregnancies:

I don’t think that you have to double your intake or anything like that. Like I was saying, I would always allow myself little treats and I would be conscious of maybe upping my calories by 200/300 a day or something you know, but just eating lots of healthy stuff and as I normally would. (Linda)

#### *The risks or rewards of physical activity in pregnancy*

Many of the women believed that pregnancy was a time for rest. Many of the women also described cutting back heavily on physical activity during pregnancy due to fears for the baby - especially in their first pregnancy:

I used to go to the gym two or three times a week before I had Anna [first child] and then when I had Anna I didn’t want to run, I didn’t want to use the weights, I didn’t want to do anything that I’d done before, because I just didn’t want to hurt her. (Celia)

Some of these women perceived that their sedentary lifestyle during pregnancy contributed to high weight gains, especially if they had a big drop in physical activity from pre-pregnancy levels:

It [exercise] went out the window [in the second pregnancy]. I suppose I thought because I was kind of so toned and so I had lost about a stone before I got married. I kind of said, I can be a bit more…give myself a bit more leeway, but I think I gave it too much. (Lisa)

In contrast, some of the women perceived that physical activity during pregnancy was healthy for the baby and strived to do as much as they could physically manage:

Yes, more movement is much, much better. Some women think it is better to stay at home, sit on the chair, watch TV - that is good for the baby. It is not, I think movement is healthier. (Caroline)

Many of the participants perceived that they 'listened’ to their body or used 'common sense’ to guide their physical activity habits during pregnancy. Engaging in gentle exercise, such as walking or pregnancy yoga was generally perceived as enough during pregnancy. All of the women who were physically active in their leisure time before pregnancy therefore halted or curtailed their strenuous physical activities during pregnancy. Some of these women swopped vigorous physical activities such as jogging for gentler exercises which they perceived as safer, such as swimming.

### Managing the home and work environment

The mothers perceived that they had much greater demands on their time in their second pregnancy, as they had to balance childcare and work responsibilities. This demanding lifestyle was perceived by the women to reduce their control over eating and physical activity habits and thus their weight. Tiredness from a long day at work and from looking after their child when they got home was perceived by some mothers to contribute to unhealthy eating habits and a sedentary lifestyle during their second pregnancy. Some of the women described using energy dense, convenience foods as a means of overcoming the tiredness which resulted from an extremely busy lifestyle:

Yeah, towards the end [of the second pregnancy] I kind of just wanted to eat a lot basically – chocolate and whatever so…I mean I worked up until 2 weeks before he was due and I think that was a bad idea anyway…I was very, very tired at that stage. Working full-time and then having to spend time with her [first child] in the evening. The weekend was the only time I got to spend full days with her – it’s actually very stressful working full time and being pregnant and trying to care for another child – it was a bit of a nightmare. (Clare)

Many of the women also noted that it was easier to do physical activity in their first pregnancy, as they didn’t have a child to mind:

I walked a lot during my first pregnancy, I didn’t walk quite as much during my second because of my little girl – I think it was easier during the first pregnancy because I could just go walking whenever, whereas I would have to wait until she was in bed you know. (Lorraine)

A supportive home environment was perceived to enable healthy prenatal lifestyle practices. A woman who developed gestational diabetes during her second pregnancy perceived that support from her partner and sister helped her to stick to her prescribed diet:

So my sister and my partner they would be the people that I spend most of my time with and so if I needed to eat they would feed the baby, or if I was eating and the child needed to be fed they would take over and they would do it, so it was quite good like that. (Sonia)

Another woman perceived that a lack of family support nearby to provide childcare was a barrier to physical activity during her second pregnancy:

To go out and exercise…that’s a big issue I think, you know, if you have children already and if you don’t have…family support, if you don’t have people that can come in for an hour or two or three to look after children you’re just not free, you know. (Cliona)

### Bodily changes

Physical conditions and physical discomforts during pregnancy were often perceived to limit women’s control over lifestyle practices and therefore weight gain. These bodily changes included nausea, heartburn, fatigue, backache, a growing body and pelvic problems. Particularly in early pregnancy, nausea led some mothers to eat less and others to eat more regularly in an effort to counteract the symptoms. In addition, many of the women found that their ability to exercise during pregnancy was constrained due to physical conditions or the physical discomforts of a growing body:

I did walk up until I was about 5 months’ [gestation in the second pregnancy]…but my hips started getting sore and my bump was getting bigger and I was just uncomfortable and I was out of breath, so I couldn’t do much. (Emma)

Furthermore, some women were advised by health professionals to stop exercising during pregnancy, because of their pregnancy complications. Finally, pregnancy conditions such as Symphysis Pubis Dysfunction (SPD) impaired some women’s mobility which adversely affected their lifestyle practices:

My circumstances during my first pregnancy, they were fine really up until about 22 or 24 weeks when I developed Pubis Symphysis disorder and I couldn’t move. That affected my eating because Patrick [husband] was working full-time, so it ended up being an awful lot of takeaways. Throughout my second pregnancy, from the very start I had the same thing and dinners were left and it ended up being takeaways. (Laura)

The availability of support from health professionals and close family members was perceived as crucial for managing and recovering from physical conditions during and after pregnancy. A mother who had SPD in both pregnancies described the importance of timely support from health professionals in her recovery:

Damien [first child] was only 9 months when I got pregnant again so I really think my body didn’t have enough time to recover properly from it. So, when I got pregnant, just right from the very start, from 8 weeks I felt my hips move. I got much better care the second time. The first time I told them from about 16 weeks that I wasn’t feeling right. I was feeling very difficult and stiff and everything when I got up to the physiotherapist. They just didn’t listen to me. When I got pregnant with Fergal [second child] I said it to them from the very start and because I had it with Damien; so because of the care I wasn’t as bad with him as I was with Damien. (Laura)

### Weight consciousness

Many of the mothers described feeling more relaxed about their weight during pregnancy than at other times. Many felt that you shouldn’t worry about your weight during pregnancy, because it is a natural part of pregnancy over which you have little control – as everybody gains weight differently. A number of women also expressed that weight gain should only be a concern if a woman’s diet is unhealthy:

I know that you need to have a good weight gain. But every woman is different to start off with. Every baby has a different weight gain. So I wouldn’t put myself under pressure or I wouldn’t want any woman to put themselves under pressure that they have to have a certain weight gain. If they are too heavy that they start dieting during pregnancy, that wouldn’t be good either. So just really have your good three meals a day and healthy snacks and even if you feel like eating out of normal, eat out of normal, but healthily of course. (Jane)

I think that if you’re eating healthily you shouldn’t worry too much about how much you gain, because everybody is different. (Laura)

Although not asked directly about their attitude towards having a high birth weight infant in their first pregnancy, it was apparent from the dialogues that most perceived big babies as healthy. Some of the women eschewed exercising normal levels of dietary restraint during pregnancy, as they perceived that high food intake and weight gain are beneficial for fetal development. In addition, any excess weight can be lost afterwards:

The best part about being pregnant [laughter] is just eating what you like and not worrying about the weight. (Sonia)

Postpartum weight retention was generally the only negative outcome of high gestational weight gain that was perceived by the women. Therefore, some of the women observed that they prioritised the health of their unborn baby over their own weight or physical appearance:

I just think people should be allowed to put on what they want when they’re pregnant and not have to worry about it too much. As long as their baby is healthy and once they are healthy…if they want to lose it after they have the baby, then they should just worry about it and do something about it if they want. (Sinéad)

It was evident from the dialogues that the experience of having a prior macrosomic delivery did *not* alter most women’s perspectives on weight gain and lifestyle practices in their second pregnancy. In contrast, one mother, who found her first childbirth experience very stressful as she had an episiotomy and had to use a crutch afterwards while it healed, decided to modify her eating habits in her second pregnancy in an effort to have a smaller baby:

I felt like I just didn’t, I didn’t want to have a big, you know a bigger baby and a lot of people had said to me oh if…your first baby is big, your second baby will be bigger and I just kind of panicked then, I thought oh Jesus I don’t want to have a bigger baby than that, so I just really watched [my eating habits] and spoke to the Consultant in Holles Street [the hospital] and she just told me things like, because I love pasta, so she said try and cut down on pasta because it’s full of sugar and little things like that. (Sarah)

Two mothers who had a lower weight gain and a smaller baby in their second pregnancy discerned health benefits of this, although neither had made deliberate attempts to avoid the recurrence of macrosomia (Caroline largely attributed her lower weight gain to staying active by working throughout her second pregnancy compared to finishing up 3 months early in her first pregnancy, whilst Miriam attributed it to heartburn which affected her eating habits):

This time I’d say I ate less, I moved more and I had the baby one week earlier and the baby was smaller, so I’d say it is more movement, less food, smaller baby and less weight…That was an easier pregnancy as well, because the belly was smaller and I didn’t put on much weight. (Caroline)

I had Symphysis Pubis Dysfunction…But it wasn’t actually so bad after the second time because I think because he [second baby] was that bit lighter. (Miriam)

The interviewees reported that the bodily changes of pregnancy diminished their ability to monitor their weight gain. One woman recalled having little awareness of how much weight she had gained during her first pregnancy, as she could no longer 'see’ her body, as it was blocked by the bump. Furthermore, some women reported that they normally used their clothes as an indicator of weight gain, rather than a weighing scale, but this indicator no longer worked during pregnancy.

Nearly all of the women were not particularly conscious or concerned about weight gain during their first pregnancy, whilst there was an increased consciousness for some women during their second pregnancy. Some of the mothers who had difficulties losing weight after their first pregnancy described being more watchful of their eating and weight gain during their second pregnancy:

I suppose I was a bit more concerned about gaining weight last time, because I think naively when I was pregnant with Liza [first child] I kind of thought the weight would just fall off me after I gave birth and it really didn’t. (Clare)

Likewise, many of those who had difficulties losing weight after their second pregnancy expressed that they would be more conscious of not gaining excessively, if they were pregnant again. This increased weight consciousness was often due to a realisation that the weight did not just 'fall off’ after having a baby.

In contrast to the rest of the women who were not particularly concerned about weight gain during pregnancy, one mother was 'horrified’ by her weight gain. She described overeating and cutting back heavily on physical activity during her second pregnancy, which contrasted with the strict weight control techniques she employed before becoming pregnant. She also hated being weighed in the hospital. When asked about her views on weight gain during pregnancy, she replied:

Dreadful, horrified, I hated going in and getting weighed in the hospital. (Lisa)

Most of the women reported not receiving any advice from health professionals about healthy eating during pregnancy, beyond which foods they should avoid. In addition, the majority of the women did not recall receiving any advice about weight gain during antenatal care. A small number of women were advised during pregnancy that their weight gain was too high. These women recounted feeling surprised, stressed, or annoyed by the comments:

The first time I was probably quite stressed because the consultant…I felt like I was going to Weight Watchers every week. (Silvia)

Furthermore, the participants were asked how they would feel if women were given a recommended weight gain range for pregnancy based on their pre-pregnancy BMI, which is standard practice in the US. This question provoked a mixed response from the mothers. Some of the women had a positive attitude towards it, as it would increase awareness about overeating during pregnancy:

Yeah, yeah, I think it is good because…I think if you knew yourself what it was, I think you might be more conscious as well. I think some people think because they’re pregnant, 'ah sure, I can eat what I want’ , and I did think like that when I was pregnant with Annette [second child] and I was kind of like, 'I’ll worry about it after’. (Lisa)

More of the mothers had a negative attitude towards it, as it would be too restrictive or stressful for women, especially overweight women:

I think that’s added pressure they don’t need during pregnancy. (Celia)

You know, people just might find it a bit irritating if it was, you know, said to you every time you went for a check-up, I don’t know larger people might not bother going for a check if they felt they were going to get a dressing down or be given out to. (Cliona)

Many of the women felt that weight gain is a personal, sensitive issue and that weight gain guidelines would be too 'clinical’. Some women felt that guidelines would need to be tailored to each individual’s circumstances, as every pregnancy is different. Some women also noted that the provision of weight gain guidelines would have to be very carefully managed to avoid putting pregnant women under undue stress. One participant, who worked as a nurse, commented:

The whole weight thing has to be carefully managed because it’s a balance between the mental health of the patient and, you know, striking a balance between that and the weight gain. (Emma)

## Discussion

This analysis exposed numerous barriers to healthy maternal weight gain, including physical conditions that impede healthy lifestyle practices, perceived risks of physical activity during pregnancy and beliefs about following all food cravings, even to excess. Indeed, the perspectives of women with a history of macrosomia are broadly compatible with those observed in previous qualitative studies on weight management during pregnancy.

Our findings are consistent with prior research showing that women are not particularly concerned about weight gain during pregnancy, as they perceive it as inevitable [36-39]. Additionally, the finding that women rarely received advice from health professionals about appropriate gestational weight gain has been reported previously [12,37,40]. A unique finding of our research was that most of the participants had a negative attitude towards the notion of introducing the Institute of Medicine recommendations for weight gain during pregnancy [1] into routine antenatal care. The mothers perceived that prenatal weight gain is different for each woman and each pregnancy and therefore weight gain recommendations would be too restrictive and stressful. Collectively, our findings suggest that if weight gain guidelines are introduced into routine antenatal care in Ireland, the individual circumstances of each pregnancy should be considered by the clinician when providing recommendations. Advice about weight gain should also be provided in a sensitive manner, as women do not feel they have much control over weight gain during pregnancy and therefore advice to restrict weight gain can cause distress.

The perception of pregnancy as an exceptional time when a women can eat whatever she wants, as a high food intake is considered to promote healthy fetal development is supported by prior research [37,40]. In addition, the perception that being pregnant is a legitimate excuse for overeating has been documented previously [41]. Most women in our research seemed unaware that they were at high risk for macrosomic delivery in their second pregnancy. Likewise, most did not alter their perspectives on weight gain and lifestyle practices in their second pregnancy as a result of having a prior macrosomic delivery. Additionally, the women seemed largely oblivious to the health risks of high weight gain beyond postpartum weight retention. On a whole, these results suggest that beliefs about the importance of high food intake for healthy fetal development are entrenched in many women’s psyche. As the women prioritised the needs of their unborn baby during pregnancy, education about the risks of excessive gestational weight gain for maternal and child health may encourage women to eat in moderation and gain a healthy amount of weight during pregnancy. In addition, it may be pertinent to provide information to pregnant women with a history of macrosomia about the high recurrence rate of macrosomia and the importance of avoiding excessive weight gain. The findings also suggest that pregnant women may need to be advised in the early stages of antenatal care to follow their food cravings in moderation.

The women recalled being much busier in their second pregnancy than their first due to the competing demands of work and childcare. However, a strong support system was perceived to motivate women to engage in healthy lifestyle practices in spite of their demanding lifestyle. Our findings suggest that women with children already may benefit from antenatal education regarding the most time-efficient methods of integrating exercise and healthy eating into their daily routines during pregnancy. Advice about physical activities that do not require a childminder should also be included. Furthermore, these findings suggest that group-based antenatal interventions may be more effective than one-to-one interventions, as repeated group contact may boost women’s motivation to adhere to an intervention and may foster women’s peer support structures.

Previous research lends support to our finding that bodily changes during pregnancy affect women’s control over weight and lifestyle practices [40,42,43]. Our research highlights that timely support from health professionls and close family members is vital for recovery from physical conditions during and after pregnancy. The research also revealed that many of the mothers cut back heavily on physical activity in pregnancy due to perceived risks to the baby, particularly in their first pregnancy. These findings suggest that pregnant women (particularly primigravids) may need education about the benefits of physical activity during pregnancy and advice about how they can modify their activities to feel safe during pregnancy.

To the best of our knowledge, this is the first study to explore perspectives on weight management during pregnancy among women with a history of macrosomia. A key strength was the use of known methods for promoting the trustworthiness of research findings [34,35], including member checking and multiple coding. A potential limitation is that the results may not be generalisable to women with a history of macrosomia who live outside of Ireland. Another potential weakness was that the mothers were interviewed 6 months after their second pregnancy, so they provided retrospective accounts of their two pregnancies which could be affected by recall difficulties or biases. Research indicates that long-term recall of perinatal information is generally accurate, although recall of maternal weight gain is poorer than other information [44]. However, the interviewees were not asked to quantify their weight gain, as this data was available in the form of serial weight measurements from the wider study. Instead, the participants provided their perspectives and experiences regarding weight gain during pregnancy - which did not require an exact knowledge of the amount of weight gained. Indeed, a potentially informative avenue for future analysis would be to divide the sample into high weight gainers and low/normal weight gainers to examine whether the responses given differed between the two groups.

## Conclusions

The analysis highlighted many barriers to healthy weight gain during pregnancy, including physical conditions, beliefs about following all cravings and perceived risks of physical activity. The research also showed that women do not favour the notion of introducing the Institute of Medicine recommendations for weight gain during pregnancy into routine antenatal care. Furthermore, the research showed that most of the women did not change their views on weight gain and lifestyle practices in their second pregnancy as a result of having a macrosomic infant in their first pregnancy. The findings have numerous implications for policy and practice. First, pregnant women may need to be advised to follow their cravings in moderation. Second, women with a history macrosomia may need advice about the importance of avoiding excessive weight gain in future pregnancies. Third, antenatal education for women with children already, should include advice on time-efficient methods of integrating healthy lifestyle practices into their daily routines. Fourth, timely support from health professionals and family members is crucial for recovery from pregnancy complications. Fifth, health professionals should advise women on how they can modify their activities to feel safe during pregnancy. Finally, health professionals and researchers may need to work in partnership with women to develop acceptable and usable solutions to the problem of excessive gestational weigth gain/macrosomia - without over-medicalising the issue, or increasing women’s stress levels during pregnancy.

## Competing interests

The authors declare that they have no competing interests.

## Authors’ contributions

EH conceived of the study, conducted the interviews and the thematic analysis and drafted the manuscript. CK contributed to the analysis and interpretation of data. PW and AM helped to design the study and contributed to the data analysis. FM participated in the study design, the data analysis and helped to draft the manuscript. All authors read and approved the final manuscript.

## Pre-publication history

The pre-publication history for this paper can be accessed here:

http://www.biomedcentral.com/1471-2393/13/202/prepub

## Supplementary Material

Additional file 1 Interview guide for the first interview. Click here for file
