# Supplementary material for: Perspectives on weight gain and lifestyle practices during pregnancy among women with a history of macrosomia: a qualitative study in the Republic of Ireland
Source: BMC Pregnancy Childbirth. 2013 Nov 6;13:202. doi: 10.1186/1471-2393-13-202 (PMC3827496; doi:10.1186/1471-2393-13-202)
Supplement: Additional file 1 — Interview guide for the first interview. [file 1471-2393-13-202-S1.docx]

**Interview guide for the first interview**

Since having your baby:

Tell me about your eating habits since having your baby?

*Probe:* *changes since having your baby?*

Tell me about your experiences of physical activity since having your baby?

*Probe: changes since having your baby?*

How do you feel about your weight since having your baby?

Tell me about:

(1) Your physical well-being? (2) Any stress experienced? (3) Support from others, if any?

During your first and second pregnancy:

[Explain that these questions refer to both their first and second pregnancy]

What do you think about eating habits during pregnancy? Own experiences?

*Probe: food cravings, nutritional changes, nausea*

What changes did you make to your eating during pregnancy?

What do you think about physical activity during pregnancy? Own experiences?

What changes did you make to your physical activity during pregnancy?

Where did you get your info about (1) diet and (2) physical activity during pregnancy?

Tell me about any advice that was given to you about weight gain during pregnancy?

What do you think about weight gain during pregnancy?

*Probe: What do you think is a healthy amount of weight to gain during pregnancy?*

How would you compare your weight gain between your first and second pregnancy?

*Probe:* *Do you think you gained a healthy amount of weight during your pregnancies?*

Are you aware of any weight gain guidelines for pregnancy? [Give explanation of the IOM guidelines]

*Probe: How would you feel if these guidelines were introduced in Ireland?*

How did you feel about gaining weight during pregnancy?

*Probe: How did you feel about your body during pregnancy?*

How did you feel about your weight just before your (1) first and (2) second pregnancy?

What do you think makes it hard for women to gain healthy weight during pregnancy? *Probe: what makes it hard for women to eat a healthy diet during pregnancy?*

Tell me about:

(1) Your physical well-being? (2) Any stress experienced? (3) Support from others, if any?

Tell me about your experiences of working outside the home during pregnancy (if any)?

*Probe: How did your work routine impact on your diet and lifestyle?*

Before having children:

Tell me about your eating habits before you had any children?

Tell me about your physical activity before you had any children?

How did you feel about your weight before you had any children?

Tell me about:

(1) Your physical well-being? (2) Your emotional well-being?

Finishing up:

Before we finish, are there any others comments you would like to make on this topic? Do you think important things were left out? Which topics?
